# Effectiveness of Telemedicine for the Management of Foot Ulcers in People With Diabetes: A Meta-Analysis

**DOI:** 10.7759/cureus.30634

**Published:** 2022-10-24

**Authors:** Ushna Khan, Khubaib Ahmad, Sai Sreya Yadlapalli, Muhammad Haseeb, Burha Kabir, Deepa Khemani, Palwasha Ghulam Moosa, Samiullah Khan

**Affiliations:** 1 Medical College, Allama Iqbal Medical College, Lahore, PAK; 2 Medical College, Service Institute of Medical Sciences, Lahore, PAK; 3 Internal Medicine, Spartan Health Sciences University, Vieux Fort, LCA; 4 Internal Medicine, Jinnah Hospital, Lahore, PAK; 5 Internal Medicine, Bahria International Hospital, Lahore, PAK; 6 Dental Surgery, Dow University of Health Sciences, Karachi, PAK; 7 Medicine and Surgery, Jinnah Sindh Medical University, Karachi, PAK; 8 Nephrology, Sindh Institute of Urology and Transplantation (SIUT), Karachi, PAK; 9 General Medicine, Baqai University, Karachi, PAK

**Keywords:** meta-analysis, ulcer healing, telemedicine, foot ulcer, diabetes

## Abstract

Treatment of diabetes-related foot ulcers presents great pressure on the healthcare system in terms of management strategy and allocation of resources. Telemedicine can be used to treat diabetic foot ulcers more effectively. This meta-analysis aims to evaluate the impacts of telemedicine on the treatment of diabetic foot ulcers. The current meta-analysis was conducted as per the reported guidelines of the Preferred Reporting Items for Systematic Review and Meta-analysis (PRISMA) statement. Two reviewers independently searched for relevant articles using PubMed, EMBASE, and the Cochrane Database of Systematic Reviews from inception to 31 August 2022, assessing the impacts of telemedicine on the treatment of diabetic foot ulcers. The primary outcomes assessed in the current meta-analysis included the percentage of foot ulcers healed and the time of healing foot ulcers within 12 months. Secondary outcomes included the percentage of amputation (minor and major) and all-cause mortality. A total of six studies were included in the current meta-analysis enrolling 1876 patients with diabetic foot ulcers. No difference was there between the two groups in terms of the number of patients whose ulcer healed (risk ratio (RR): 1.01, 95% confidence interval (CI): 0.93-1.09), time to healing of wound within 12 months (mean difference: -0.07, 95% CI: -0.31-0.17), the incidence of amputation (RR: 0.73, 95% CI: 0.54-1.00), and all-cause mortality (RR: 0.99, 95% CI: 0.42-2.37). In conclusion, the study found that telemedicine is non-inferior to standard care in terms of reducing healing time and the number of patients with ulcer healing within 12 months. The study also found that the incidence of amputation is also lower in patients assigned to the telemedicine group compared to patients in the control group and no significant differences were reported in terms of mortality.

## Introduction and background

Treatment of diabetes-related foot ulcers presents great pressure on the healthcare system in terms of management strategy and allocation of resources [1]. Nearly 7% to 15% of people with diabetes will have at least one-foot ulcer during a lifetime [2]. Diabetes is associated with neuropathy, ischemia, and deformities that lead to a greater risk of developing foot ulcers [3]. These ulcers impair the quality of life and mobility of patients and bring them great physical discomfort [3]. The presence of a foot ulcer is linked to a greater incidence of all-cause mortality [4]. Every year, 500 major amputations caused by diabetic foot ulcers cost Denmark in terms of heavy financial burden [5]. In 1995, it was calculated that a major amputation would cost between $59,000 and $87,000 [6].

Elderly individuals with comorbidities like diabetes and foot ulcers place a challenge on healthcare systems all over the world. A chronic wound is linked with increased individual psychological and physical strain and other comorbidities that further reduces their capacity to see a specialist, who, in certain regions, may be located in distant areas [7]. Finding methods to treat persons with lower limb ulcers in geographically remote areas is a top priority due to the lack of professionals in such fields [8]. Due to this, there is a growing focus on discovering technical solutions to enhance healthcare results without doing more harm. Telemedicine is a field that has the capability to provide quality services from a distance [9]. It includes different kinds of technologies for monitoring, treating, and developing innovative solutions and has a greater potential to enhance patients’ outcomes at a low cost [10].

Treatment of diabetic foot ulcers often needs frequent consultation and contact with health care professionals that may insert a heavy burden on patients [11]. In addition, the treatment of diabetic ulcers is challenging for healthcare professionals, as it may take several months to heal and cause amputation, gangrene, and osteomyelitis [12]. As per international guidelines, patients with diabetic foot ulcers need to be referred to specialist healthcare facilities at an early age [13]. However, in different countries, many foot ulcer patients are treated in primary care with a lack of doctors and nurses and access to special healthcare facilities [14]. Telemedicine can decrease the demand for specialist healthcare professionals by transferring follow-up and treatment to home or primary healthcare facilities while sustaining a high quality of wound care. These characteristics imply that telemedicine, when offered by a multidisciplinary team via an interactive platform, has the promise to be a successful strategy in the management of diabetic foot ulcers in patients with diabetes [12].

There is an increasing interest in reducing outpatient consultations and hospital visits and easing the care of individuals with diabetes and foot ulcers, particularly in remote areas. For more efficient management of diabetic foot care, telemedicine can be a solution to enhance communication among different levels of care and increase the quality of care in health care services [12]. However, the efficiency of telemedicine interventions for patients with diabetic foot ulcers related to organizational, behavioral, and clinical outcomes in comparison to traditional follow-up care is unclear. In order to evaluate the function of telemedicine for this illness, a high-quality systematic review is required because the apparent effects of telemedicine for the treatment of diabetic foot ulcers are still up for debate. This meta-analysis aims to evaluate the impacts of telemedicine on the treatment of diabetic foot ulcers.

## Review

Methodology

The current meta-analysis was conducted as per the reported guidelines of the “Preferred Reporting Items for Systematic Review and Meta-analysis” (PRISMA) statement.

Search Strategy and Study Selection

Two reviewers independently searched for relevant articles using PubMed, EMBASE, and the Cochrane Database of Systematic Reviews from inception to 31 August 2022, assessing the impacts of telemedicine on the treatment of diabetic foot ulcers. The key terms used to search for relevant articles included: “telemedicine”, “diabetic foot ulcers”, “telephone”, “video consultation”, “foot ulcer” and “diabetes”. The reference list of included papers was also hand-searched for additional studies.

Abstracts and titles were screened independently against eligibility criteria followed by a full-text screening of qualifying papers. Disagreements between two authors at all stages were resolved through discussion or the involvement of a third author if required. We included all studies that assessed the impacts of telemedicine on the treatment of diabetic foot ulcers. We included studies that assessed the impacts of telemedicine on patients of any age with diabetes mellitus and related foot ulcers. We considered all kinds of telemedicine for this meta-analysis, including internet-based two-way video conferencing, telediagnostic services, and interactive consultations. We excluded cross-sectional studies, case reports, reviews, and case series from the current meta-analysis.

Outcomes

The primary outcomes assessed in the current meta-analysis included the percentage of foot ulcers healed during the study period. The second primary outcome was the time of healing of foot ulcers (in months). Secondary outcomes included the percentage of amputation (minor and major) and all-cause mortality.

Risk of Bias Assessment

The risk of bias assessment of all studies was done by two authors independently using the Newcastle‐Ottawa Scale for cohort studies and the Cochrane bias risk assessment tool for randomized clinical trials (RCT).

Data Extraction

Data were extracted from included studies using the pre-designed data extraction form created on Microsoft Excel (Microsoft Corporation, Redmond, WA). Data extracted included author name, year of publication, study design, sample size, follow-up period, and outcome measures. Data extracted were entered into RevMan software by one author and double-checked by the second author.

Statistical Analysis

Review Manager Version 5.4.0 (The Nordic Cochrane Centre, The Cochrane Collaboration, Copenhagen) was used for data analysis. For dichotomous outcomes, data were pooled using the random effects model, according to the Mantel-Haenszel model to compute risk ratio (RR) with a 95% confidence interval (CI). For continuous outcomes, the mean difference was calculated using the random effects model along with their 95% CI. A cut-off of the p-value was kept at 0.05. Heterogeneity was calculated for each outcome using the I-square statistics. I-square values of less than 25% were classified as low, 25-50% as moderate, and more than 50% as high degrees of heterogeneity.

Results

**Figure 1:**
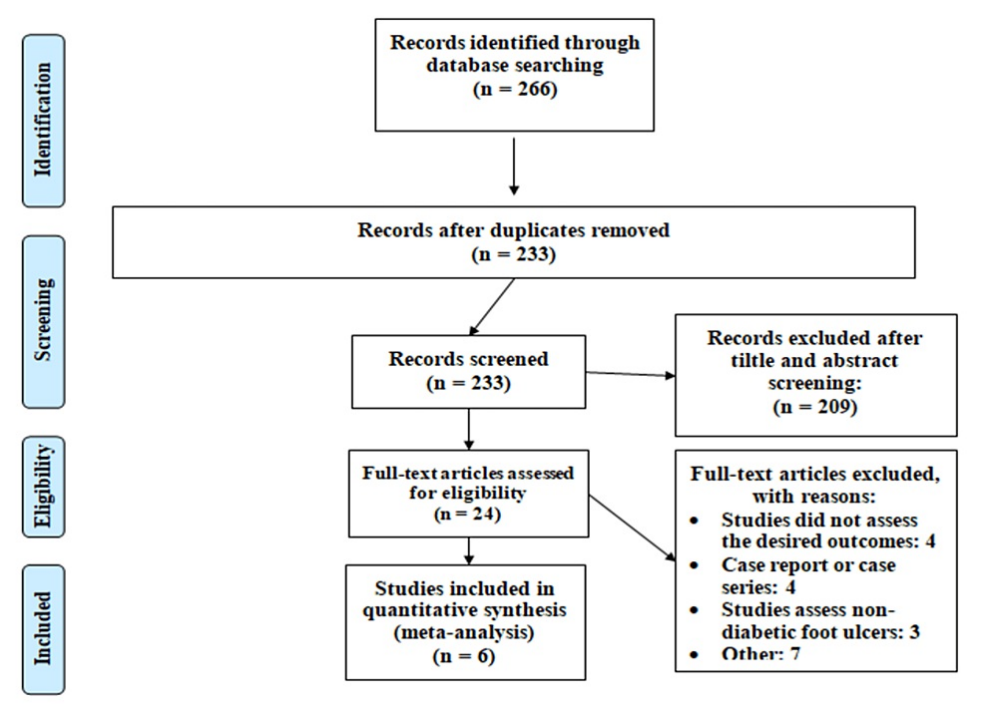
PRISMA flowchart of selection of studies PRISMA: Preferred Reporting Items for Systematic Review and Meta-Analysis

The results of the literature search and process of study selection are shown in Figure 1. An online search led to 266 articles, of which 233 articles were screened for the title and abstract screening after removing duplicates. The full text of 24 articles was retrieved to assess for inclusion and exclusion criteria. A total of six studies were included in the current meta-analysis enrolling 1876 patients with diabetic foot ulcers. Characteristics of the included studies are shown in Table 1.

**Table 1:** Characteristics of the included studies RCT: randomized control trial; * n (%)

Author	Year	Study design	Groups	Sample size	Follow-up	Mean age (Years)	Males*
Fasterholdt et al [3]	2018	RCT	Telemedicine	191	6 Months	NR	NR
			Control	181			
Rasmussen et al [9]	2015	RCT	Telemedicine	193	12 Months	66.7	280 (44.8)
			Control	181			
Rastogi et al [14]	2021	Observational	Telemedicine	259	6 Months	56	NR
			Control	366			
Smith-Strom et al [15]	2018	Clustered RCT	Telemedicine	94	12 Months	66.4	135 (74.2)
			Control	88			
Teot et al [16]	2019	RCT	Telemedicine	89	6 Months	72.5	89 (48.6)
			Control	94			
Wilbright et al [17]	2004	Non-randomized trial	Telemedicine	20	3 Months	55.8	65 (46.4)
			Control	120			

**Figure 2:**
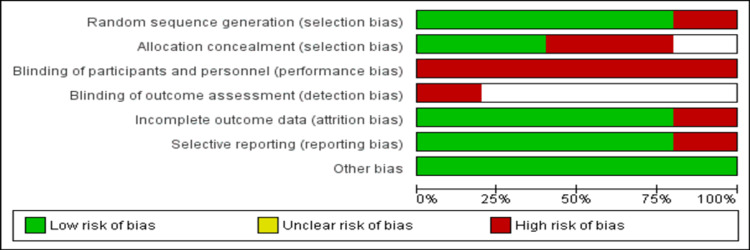
Risk of bias assessment of clinical trials

Among all included studies, four were randomized control trials [3,9,15,16], while one study was an observational cohort [14] and one was a non-randomized study [17]. The pooled mean age of patients was 63.4 years. Figure 2 shows the risk of bias assessment of all clinical trials and Table 2 shows the quality assessment of the observational study. The overall quality of the study was moderate considering that blinding of participants was not possible in trials assessing the impact of telemedicine in comparison with standard care.

**Table 2:** Quality assessment of observational study

Study ID	Selection	Comparability	Outcome	Overall quality
Rastogi et al, 2021 [14]	4	2	3	Good

**Figure 3:**
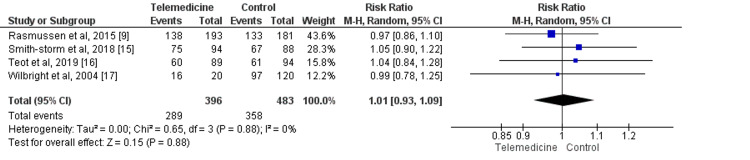
Forest plots showing the risk ratio (RR) of the number of patients whose ulcers healed Sources: References [9,15-17]

Four studies compared the number of patients, whose ulcers healed, with a total of 879 patients who were either in the telemedicine group or the control group [9,15-17]. No difference was there between the two groups in terms of the number of patients whose ulcer healed (RR: 1.01, 95% CI: 0.93-1.09) as shown in Figure 3. Statistical heterogeneity was assessed as low in the pooled effect (I-square: 0%).

**Figure 4:**
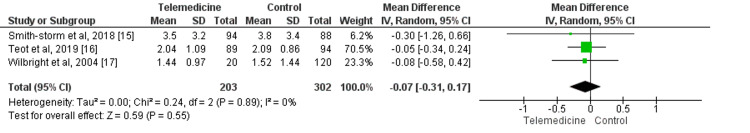
Forest plot of time of healing of foot ulcers Sources: References [15-17]

Three studies examined the timing of healing of foot ulcers, with a total of 505 patients [15-17]. We found no significant difference in the time of healing in months between the two groups between the telemedicine group and the control group (mean difference: -0.07, 95% CI: -0.31-0.17) as shown in Figure 4. Statistical heterogeneity was assessed as low in the pooled effect (I-square: 0%).

**Figure 5:**
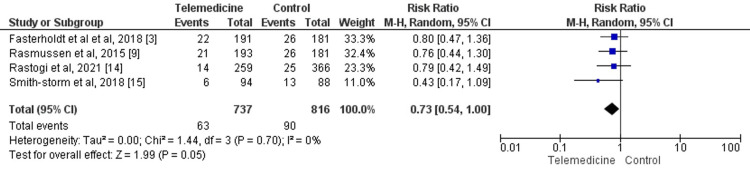
Forest plot of reduction of amputation Sources: References [3,9,14-15]

Four studies compared the incidence of amputation between both groups, a total of 1553 patients [3,9,14-15]. The incidence of amputation was significantly lower in patients in the telemedicine group compared to the control group (RR: 0.73, 95% CI: 0.54-1.00) as shown in Figure 5. Statistical heterogeneity was assessed as low in the pooled effect (I-square: 0%).

**Figure 6:**
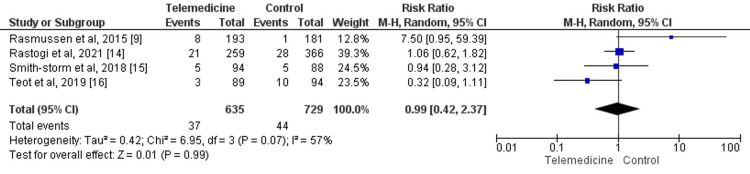
Forest plot of all-cause mortality Sources: References [9,14-16]

Four studies assess the risk of all-cause mortality between both groups including a total of 1364 patients. The incidence of mortality was not statistically significant in the telemedicine group and control group (RR: 0.99, 95% CI: 0.42-2.37) as shown in Figure 6. Statistical heterogeneity was assessed as moderate in the pooled effect (I-square: 57%).

Discussion

This meta-analysis aims to discuss and review the efficiency of telemedicine in the care of patients with diabetic foot ulcers. The current meta-analysis found that telemedicine is as good as standard care in attaining healing time and ulcer healing. The study also found that the incidence of amputation is lower in patients assigned to the telemedicine group as compared to patients in the control group. The current meta-analysis shows that telemedicine is not inferior to usual standard care.

Our study found a similarity in the healing rates between the two groups that may be attributed to enhanced access to trained healthcare professionals instead of the utilization of telemedicine technology per se. However, telemedicine also makes rapid access to medical professionals and interdisciplinary foot care specialists possible, considerably accelerating the delivery of care and solving treatment issues that were previously sent to diabetic foot practitioners. Telemedicine consultations can enhance the ability of nurse specialists to see patients in a remote setting. Regarding the number of patients whose foot ulcers healed, all studies assessed this outcome in the current meta-analysis concluded that no significant differences are there between the two groups. However, the results need to be interpreted with caution as the sample size enrolled is quite low. In the future, RCTs with sufficient follow-up time and larger sample sizes are required to produce valid evidence related to the efficiency of follow-up care of diabetic foot and leg ulcers. Although healthcare services have utilized telemedicine for the last many years, studies that assess the impact of telemedicine have been restricted to small sample sizes and conducted over a short period [18].

The overall results of this meta-analysis point to significant conditions to consider for further implementation and use of telemedicine technology. Utilizing new technology results in adjustments to how health care is delivered as well as to how everyday labor is structured. Strategic utilization of telemedicine can help healthcare professionals to deliver integrated healthcare services across various levels of healthcare and decrease the burden on outpatient clinics. The current meta-analysis has shown that telemedicine follow-up is non-inferior in regard to foot ulcer healing time.

Current knowledge of the impacts of telemedicine has been based on low-powered studies [19] and many clinical trials related to ulcers have emphasized healing as the main outcome without even considering other outcomes like mortality and amputation. Due to the significant risk of amputation linked with diabetic foot ulcers and the fact that 85% of patients who get an amputation first have a diabetic foot ulcer [20]. Data on this, as well as other risks associated with diabetic foot ulcers, need to be gathered when assessing new interventions.

Telemedicine care for the management of diabetic patients has been in practice for two decades, but not many efforts have been made to the implementation of this system for the care of diabetic foot ulcers particularly [21]. Telemedicine is a desirable alternative due to a variety of factors in addition to its efficacy. Hospital staff and patients can readily access telemedicine, which is practical, low-cost, and operates with fewer staff members [22]. Different studies have also shown that telemedicine significantly enhances the quality of care and patient engagement as several activities are automated [22-23].

Adverse effects and safety issues are vital aspects to take into account when assessing the efficiency of telemedicine, including patient follow-up. Despite the benefits and promises that telemedicine is capable of delivery, certain challenges are there at legal, technical, and patient levels [24]. When compared to the customary face-to-face, in-the-same-room interactions between patients and healthcare providers, telemedicine may, for instance, change that relationship between patients and healthcare providers [25]. In addition, a lack of competence among healthcare practitioners deters effective execution and might cause adverse events among patients. Another important issue with telemedicine is the need for system security to guard against unauthorized access to the data. Legal requirements are crucial, but they must strike a balance between security and ease of execution [25]. Adverse events and safety issues were not reported by the included studies. These aspects are important to be highlighted in future studies.

The current meta-analysis has certain limitations. First, the majority of the included studies in the current meta-analysis had a small sample size and utilized single arm design reducing the confidence in generalizing the results to the wide population. Second, the telemedicine system is complex, and both the patients and clinical staff require time to utilize it efficiently. The follow-up duration of included studies is shorter, thus long-term outcomes could not be assessed. In the future, more studies need to be conducted that include large sample sizes and longer follow-up durations to provide more robust and generalizable results about the impact of telemedicine in patients with diabetic foot ulcers.

## Conclusions

In conclusion, the study found that telemedicine is non-inferior to standard care in terms of reducing healing time and the number of patients with ulcer healing within 12 months. The study also found that the incidence of amputation is also lower in patients assigned to the telemedicine group compared to patients in the control group and no significant differences were reported in terms of mortality. In the future, more rigorous and larger studies are required to enable strong clinical recommendations and conclusions to be made.
